# Quantitative phosphoproteomics reveals diverse stimuli activate distinct signaling pathways during neutrophil activation

**DOI:** 10.1007/s00441-022-03636-7

**Published:** 2022-05-27

**Authors:** Pooja Yedehalli Thimmappa, Aswathy S. Nair, Mohd. Altaf Najar, Varshasnatha Mohanty, Shamee Shastry, Thottethodi Subrahmanya Keshava Prasad, Manjunath B. Joshi

**Affiliations:** 1 Department of Ageing Research, Manipal School of Life Sciences, Manipal Academy of Higher Education, Manipal, 576104 India; 2 Center for Systems Biology and Molecular Medicine, Yenepoya Research Centre, Yenepoya (Deemed to Be University), Mangalore, 575020 India; 3 Department of Immunohematology and Blood Transfusion, Kasturba Medical College, Manipal, Manipal Academy of Higher Education, Manipal, 576104 India

**Keywords:** Neutrophils, Type 2 diabetes, Hyperglycemia, Homocysteine, Infections

## Abstract

Neutrophils display functional heterogeneity upon responding diversely to physiological and pathological stimulations. During type 2 diabetes (T2D), hyperglycemia constitutively activates neutrophils, leading to reduced response to infections and on the other hand, elevated metabolic intermediates such as homocysteine induce bidirectional activation of platelets and neutrophils leading to thrombosis. Hence, in the context of T2D-associated complications, we examined the influence of high glucose, homocysteine, and LPS representing effector molecules of hyperglycemia, thrombosis, and infection, respectively, on human neutrophil activation to identify distinct signaling pathways by quantitative phosphoproteomics approach. High glucose activated C-Jun-N-Terminal Kinase, NTRK1, SYK, and PRKACA kinases associated with Rho GTPase signaling and phagocytosis, whereas LPS induced AKT1, SRPK2, CSNK2A1, and TTN kinases involved in cytokine signaling and inflammatory response. Homocysteine treatment led to activatation of  LRRK2, FGR, MAPK3, and PRKCD kinases which are associated with neutrophil degranulation and cytoskeletal remodeling. Diverse inducers differentially modulated phosphorylation of proteins associated with neutrophil functions such as oxidative burst, degranulation, extracellular traps, and phagocytosis. Further validation of phosphoproteomics data on selected kinases revealed neutrophils pre-cultured under high glucose showed impeded response to LPS to phosphorylate p-ERK1/2^Thr202/Tyr204^, p-AKT^Ser473^, and C-Jun-N-Terminal Kinase^Ser63^ kinases. Our study provides novel phosphoproteome signatures that may be explored to understand neutrophil biology in T2D-associated complications.

**Supplementary information:**

The online version contains supplementary material available at 10.1007/s00441-022-03636-7.

## Introduction

Neutrophils representing major cell types of the innate immune system are the most abundant leukocytes in circulation and actively participate in acute and chronic inflammation. Mounting pieces of evidence indicate neutrophils display a large phenotypic heterogeneity and functional versatility, as important modulators of both inflammation and immune responses rather than a homogenous population of terminally differentiated cells with a unique function (Rosales 2018). Effector functions of neutrophils to combat pathogens include (a) degranulation and oxidative burst, (b) phagocytosis, and (c) producing extracellular traps. Neutrophils upon activation with a wide variety of pathogens such as bacteria, viruses, protozoans, and helminths lead to expulsion of DNA bound with histones and granular proteins to form extracellular traps (NETs) (Brinkmann et al. 2004; Díaz-Godínez and Carrero 2019). High concentrations of antimicrobial effectors within these DNA lattices serve as a platform to activate pro-inflammatory mediators, immobilize and kill the pathogens and simultaneously clear the infection. However, besides the active role in acute infections to eliminate pathogens, externalized chromatin and components of NETs contribute to the pathogenesis of diseases associated with sterile inflammation including vascular disorders, autoimmune diseases, digestive disorders, type 2 diabetes (T2D), and cancers (Kaplan and Radic 2012; Papayannopoulos 2018).

In the context of T2D, accumulating evidence from many studies including from our own group have demonstrated abnormal phagocytosis, degranulation, and NET formation and subsequently leading to either reduced response to pathogens or for the pathogenesis of vascular complications and delayed wound healing as functional consequences of neutrophil dysfunction (Joshi et al. 2013; Menegazzo et al. 2015; Yang et al. 2020). High glucose facilitated the formation of NADPH oxidase-dependent constitutive and weak NETs and further rendering neutrophils to respond weakly to LPS in T2D subjects (Joshi et al. 2013). Hyperglycemia induced metabolic reprogramming in neutrophils and increased polyol pathway intermediates along with a reduction in antioxidants and led to deficiency of NADPH, a pre-requisite to produce extracellular traps resulting in decreased response to LPS (Joshi et al. 2020). Our studies in T2D subjects, also revealed homocysteine treatment led to bidirectional activation between neutrophils and platelets to induce NETosis and platelet aggregation, and this process was accelerated by hyperglycemic conditions (Joshi et al. 2016). Further evidences suggested a positive correlation between T2D-associated vasculopathies (nephropathy and cardiovascular diseases) and the elevated plasma levels of NET components such as cell-free DNA (cfDNA), nucleosomes, and neutrophil elastase (De Meyer et al. 2012). Wong et al. (2015) demonstrated the delayed wound healing in diabetic mouse models in Pad4-dependent manner as a consequence of hyperglycemia primed NETs (Wong et al. 2015). Impaired neutrophil migration to airways in response to LPS was observed in T2D models of Goto-Kakizaki (GK) rats. GK rats exhibited a reduction in chemokines and cytokines such as IL-1β and TNF-α concentration and lower expression of LFA-1 and ICAM-2, on neutrophils (Kuwabara et al. 2017). Higher concentrations of intracellular calcium and reduced ATP levels associated with hyperglycemia inhibited the phagocytic ability of PMN cells, and neutrophil function was restored upon glycemic control (Schuetz et al. 2011). Hair et al. (2012) demonstrated that elevated glucose and its interaction with complement system activation to opsonic forms led to reduced phagocytosis and bacterial killing by neutrophils in response to *S. aureus* (Hair et al. 2012). Stegenga et al. (2008) observed impaired neutrophil degranulation in 8 h post-treatment induced hyperglycemia of healthy individuals challenged with bacterial endotoxin (Stegenga et al. 2008). In response to *Burkholderia pseudomallei*, diabetic subjects displayed a significant reduction in the formation of NETs, phagocytosis, and chemotaxis resulting in reduced bacterial elimination (Gan 2013). Taken together, neutrophils display paradoxical effects and play beneficial role in eliminating pathogens in healthy individuals and adverse effects in the T2D microenvironment where deranged immuno-metabolic axis primes neutrophils leading to their functional impairment.

Over functioning of neutrophils leads to several off-target effects during the pathogenesis of T2D and associated complications. Hyperglycemia induces constitutive formation of weaker and dysfunctional NETs and impedes further response of neutrophils during infections. On the other hand, in T2D, metabolic intermediates such as homocysteine also stimulate neutrophils and further facilitate activation of endothelial cells and platelets leading to a conducive environment for thrombosis. Hence, inhibiting glucose and homocysteine-induced NETs and simultaneous activation of neutrophils to respond to infections may serve as a therapeutic strategy for clinical management of T2D-associated infections. To address this, we explored the phosphoproteomics approach to understand if distinct signaling pathways are activated by three different inducers, high glucose, homocysteine, and LPS representing hyperglycemia, thrombosis inducer, and infection, respectively. Further, we examined how these varied activators alter phosphorylation of proteins involved in diverse neutrophil-mediated biological processes such as (a) chemotaxis, (b) degranulation, (c) respiratory burst, (d) phagocytosis, and (e) NETosis.

## Methods

### Recruitment of subjects

We recruited seven healthy voluntary blood donors (all males of 25–30 years old) visiting the department of Immunohematology and Blood Transfusion of a tertiary care center upon approval of the study protocol from the institutional ethics committee, Kasturba Hospital, Manipal Academy of Higher Education, Manipal, India (374/2018). Donors were screened and selected for blood donation as per Directorate of General for Health Services (DGHS) guidelines. We obtained prior informed written consent from the donors to utilize the buffy coat sample for isolation of neutrophils.

### Neutrophil isolation

The buffy coat derived during the separation of blood components from a blood unit was collected in a top, and bottom quadruple bag was used as a source for neutrophils. Neutrophils were isolated using the Ficoll-Dextran method (Joshi et al. 2020). Further, neutrophils were processed by washing and re-suspending in Hanks’ Balanced Salt Solution (HBSS) with a supplement of 5-mM glucose. Quality of neutrophil was determined by Leishman’s staining and flow cytometry-based CD16 staining. Trypan blue assay was performed as routine analysis to examine the viability of cells. We observed 90–95% of viable cells at the beginning of the experiment, and approximately 10–15% of cell death was observed after 30 min of treatment. Cells were re-suspended in RPMI 1640 medium (Himedia, Mumbai, India) which included 1% heat-inactivated human serum (Invitrogen) and L-glutamine (2 mM) and subsequently subjected to various biochemical assays.

### Quantitation and imaging of NETs

Neutrophils isolated from peripheral blood were seeded at the density of 10^5^ cells/100 μL/well in 96 well plates and activated with different stimuli as indicated in figure legends. Lipopolysaccharides (LPS), glucose, and homocysteine (Hcy) were purchased from Sigma St Louis, MO, USA. Three hours post-treatment, DNA lattices were stained with SYTOX green nucleic acid stain (Thermo Fisher Scientific, MA, USA), and fluorescence was quantified in Varioskan Flash (Thermo Fisher Scientific, MA, USA). Fluorescence microscopy images of extracellular traps were captured under Olympus IX51 microscope conjugated with Rolera EM-C2 camera. Images were analyzed with image-Pro Plus software v7.0.

### Measurement of neutrophil elastase levels

Neutrophils were seeded at the density of 10^6^ cells/mL and treated with different activators as indicated in figure legends. After incubation, conditioned medium was subjected to elastase measurement using human neutrophil elastase ELISA kit (Duoset, Biotechne, R&D systems). Plates coated with carrier proteins were incubated for 120 min with 100µL of standards and samples followed by repeated wash to remove unbound antigen. Samples were then incubated with detection antibody conjugated with streptavidin-HRP and substrate solution respectively as per the manufacture’s instructions. Optical density was measured immediately after the addition of stop solution at 450 nm with a wavelength correction of 540 nm in Tecan infinite 200 microplate reader (Thermo Fisher Scientific, MA, USA).

### Immunoblotting

Neutrophils (2 × 10^6^ cells/mL) were activated with diverse stimuli in HBSS buffer as described in figure legends. Further, cells were collected by centrifugation at 1000 RPM for 10 min and lysed using RIPA buffer [50-mM Tris pH 7.4, 15-mM NaCl, 5-mM EGTA, 0.1% SDS, 1% Sodium deoxycholate, 1% NP-40, 10-mM sodium pyruvate, 0.5-mM sodium fluoride and protease inhibitor cocktail (Roche, Switzerland)]. Equal concentrations of proteins were separated on the 10% denaturing polyacrylamide gel and blotted onto nitrocellulose membranes. The membranes were further probed using respective primary antibodies. Anti-citrullinated histone (Abcam, Cambridge, UK), Anti-p^44/42^ ERK (Thr202/tyr204), Anti-ERK, Anti-pAKT (Ser473), Anti-AKT, Anti-p–C-JUN (ser 63), and Anti-C-JUN (all from Cell Signaling Technology, Massachusetts, USA) were hybridized for 1 h at room temperature and followed by horseradish peroxidase-conjugated secondary antibodies (Thermo fisher, US) for another 1 h at room temperature. Immunoblots were developed using enhanced chemiluminescence kit (ECL) (Pierce, Thermo Fisher Scientific, MA, USA) and imaged in ImageQuant Las 4000. Images of immunoblots were processed using ImageJ software (NIH, USA) for densitometric analysis.

### Sample preparation for phosphoproteomics analysis

#### Protein isolation and digestion

Neutrophils were treated with high glucose (25 mM), LPS (2 μg/mL), and homocysteine (250 μM) for 30 min, and protein extraction was performed as discussed previously (Najar et al. 2021b). Briefly, cells were lysed using lysis buffer containing 2% SDS in 50-mM TEABC along with 1-mM sodium orthovanadate, 2.5-mM sodium pyrophosphate, and 1-mM β glycerol phosphate and followed by centrifugation for 30 min at 14,000 rpm at 4 °C. Protein concentrations were measured in supernatants by the BCA method (Pierce, Waltham, MA) and were also confirmed visually upon separation of proteins on 10% SDS–polyacrylamide gel electrophoresis (PAGE) gel.

#### Trypsin digestion and fractionation

Cell lysates obtained from different individuals upon treatment were pooled, and 150 µL of cell lysates from each condition (total concentration:420 µg) were processed using modified filter-aided sample preparation (FASP) protocol established by Verma et al. (2017). In brief, the cell lysates were treated with 5-mM dithiothreitol (DTT) and 20-mM iodoacetamide to reduce and alkylate respectively. Further, the SDS concentration was reduced to 0.001% using 8-M urea. Before subjecting samples to trypsin digestion, urea was removed by exchanging buffer with the 50-mM TEABCC. The samples were digested with trypsin (1:20) (Worthington Biochemical Corp.), overnight at 37 °C, and vacuum-dried, and peptides were stored at − 80 °C until further analysis.

#### Tandem mass tag (TMT) labeling for phosphoproteome quantitation

Peptides extracted from cells treated with different inducers were reconstituted in 50-mM TEABC (pH 8.0) prior to labeling with 10-plex TMT (Thermo Scientific, Bremen, Germany). Reconstituted peptides were split into three parts to serve as technical replicates. TMT labeling was performed as indicated: untreated neutrophils were labeled with channels 127 N and neutrophils which are treated with high glucose, homocysteine and LPS were labeled with 128 N, 129 N, and 130 N, respectively, as per the manufacturer’s protocol. Hydroxylamine (8 μL of 5%) was added and incubated at room temperature for 15 min for quenching the reaction and dried (Verma et al. 2017). Equal amount of labeled peptides were pooled after performing a TMT label check followed by vacuum drying before phosphopeptide enrichment.

#### TiO2-based phosphopeptide enrichment and basic pH RPLC (bRPLC)

For phosphopeptide enrichment 90% of the TMT labeled peptides were used, and phosphopeptide enrichment using TiO2 was performed as described previously (Najar et al. 2021a, b). Remaining one-tenth volume from each fraction was used for the total proteome analysis. TMT-labeled peptides were resuspended in 2,5-dihydroxybenzoic acid (DHB) solution (5% 2,5-dihydroxybenzoic acid, 80% ACN, 3% TFA, HPLC grade). TiO2 beads (GL Science 5020–75,010) were dehydrated and further resuspended in 5% DHB solution and followed by incubation at room temperature for 15 min on the rotator. Subsequently, each sample was mixed with an equal volume of beads and was incubated at room temperature for 1 h on a rotator. Further, beads were spin down, followed by stepwise washing with wash solution 1 (80% ACN, 3% TFA, HPLC grade), wash solution 2 (80% ACN, 1% TFA, LCMS grade), and wash solution 3 (80% ACN, 0.1% TFA, LCMS grade). Finally, elution solution (4% NH_4_OH, LCMS grade water) was used to elute phosphopeptides in a microfuge tube containing 10 µl of 3% formic acid on ice and vacuum dried using a vacuum concentrator and stored at − 20^o^ C.

Fractionation of phosphopeptides labeled with 10-plex TMT was performed using high pH reverse phase LC (Verma et al. 2017). In brief, the resuspension of labeled peptides in bRPLC solvent (10-mM TEABC pH 8.4) and high pH reverse phase XBridge C18 column (5 μm, 250 × 4.6 mm^2^) (Waters Corporation, Milford, MA) used for fractionation by employing an increasing gradient of bRPLC solvent B (10-mM TEABC in 90% ACN, pH 8.4) using Agilent 1100 LC system with a flow rate of 1 mL/min. A total of 96 fractions were collected in a 96-well plate containing 0.1% formic acid, and the fractions were then concatenated to 6 fractions and vacuum dried.

#### LC–MS/MS analysis

Mass spectrometry data was acquired in Orbitrap Fusion Tribrid mass spectrometer (Thermo Fischer Scientific, Bremen, Germany) coupled to Easy-nLC-1200 nanoflow liquid chromatography system (Thermo Scientific). The peptides were reconstituted in 0.1% formic acid and loaded onto a 2-cm trap column (nanoViper, 3 µm C18 Aq) (Thermo Fisher Scientific) and resolved using a 15-cm analytical column (EASY-Spray column PepMap RSLC, C18, 2 µm, 100A, 75 µm × 15 cm) at a flow rate of 300 nL/min. The solvent B set for a gradient of 535% (80% acetonitrile in 0.1% formic acid) for 90 min and a total run time of 120 min for each fraction in triplicates. Global MS survey scan at a range of 4,001,600 m/z mass range (120,000 mass resolution at 200 m/z) in a data-dependent mode using an Orbitrap mass analyzer was carried out. Peptides with charge states 26 were considered for analysis, and the dynamic exclusion rate was set to 30 s. For MS/MS analysis, precursor ion fragmentation was performed using higher collision energy dissociation with 34% normalized collision energy. MS/MS scans were carried out at a range of 110-2000 m/z using an Orbitrap mass analyzer at a resolution of 60,000 at 200 m/z. The raw data acquired were processed using Proteome Discoverer software suite version 2.2 (Thermo Fisher Scientific); the data was searched against RefSeq 94 database. Protein sequences were downloaded from NCBI, and MS/MS data were searched against the protein database along with known mass spectrometer contaminates using the SEQUEST algorithm. Search parameters were fixed as carbamidomethylation of cysteine as a static modification, oxidation of methionine, deamination, and N-terminal acetylation at protein N-terminus and minimum peptide length of seven amino acids with 1 missed cleavage. Mass tolerance was set to 10 ppm at MS level and 0.05 Da for MS/MS.

#### Statistical and bioinformatics analysis

Statistical analysis was performed using GraphPad Prism 8.0.2. Multiple *t* test was performed between the groups and *p* values less than 0.05 were considered statistically significant. Gene ontology (GO) terms, biological components, and signaling pathways were identified by performing gene enrichment analysis using Enrichr online tool and Reactome pathways online databases. Protein–protein interaction networks were retrieved by the STRING database (version 11). The kinase-substrate prediction analysis of phosphopeptides was validated using K3 kinase enrichment analysis, version 3. Heat maps and Venn diagrams were generated using Morpheus online tool and Microsoft excel, respectively. Abundance values of phosphorylated proteins in neutrophils in response to high glucose, homocysteine, and LPS compared with control were used to represent graphically using CIRCOS.

## Results

### Neutrophils display intra-individual variations to form extracellular traps in response to diverse inducers

**Fig. 1:**
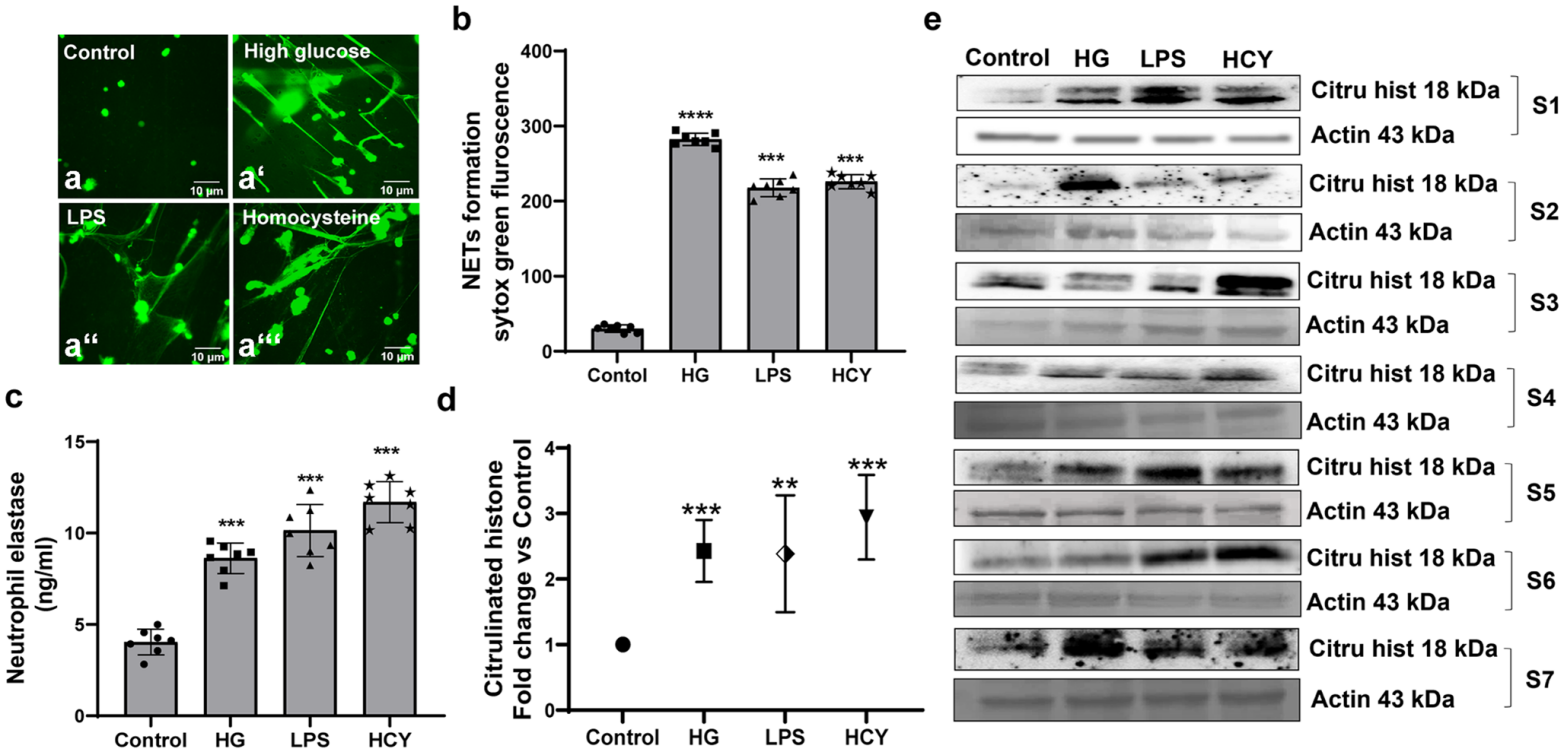
Neutrophils display varied responses to different inducers to form extracellular traps. Neutrophils isolated from peripheral venous blood from healthy donors (*n* = 7) were stimulated with high glucose (25 mM), LPS (2 µg/mL), and Hcy (250 µM) for 3 h (a, a’, a’’, a’’’, b & c). Extracellular DNA was stained with SYTOX green (**a**, **a’**, **a’’**, **a’’’**). Representative images of NETs induced by different activators captured using a fluorescence microscope are provided. Scale bar 10 μm. (**b**) Fluorescence intensity of SYTOX green was measured at 480/530 nm and NETs were quantified. Data is represented as a percentage of maximum fluorescence. (**c**) Supernatants from neutrophils treated as above were collected and elastase levels were measured. (**d**) The neutrophil lysates upon treatment with inducers for 30 min were subjected for immunoblotting. Representative blots stained for citrullinated histones and actin (Ponceau) are shown. (**e**) Data are represented as fold change Vs control. Statistically significant values obtained for all experiments are indicated by an asterisk *p < 0.05, **p < 0.001, ***p < 0.0001

As a first step, we examined the magnitude of NETs formation in response to diverse inducers by various cellular and biochemical assays. Fluorescence imaging and neutrophil elastase assays, in neutrophils cultured in presence or absence of high glucose (HG-25 mM), lipopolysaccharides (LPS-2 µg/ml), and homocysteine (Hcy-250 µM), for 3 h revealed that all these three stimulators displayed similar effects on neutrophils to form NETs. High glucose facilitated a nearly sixfold increase in NET formation, whereas homocysteine and LPS treatment produced 5–6 folds of NETs compared to untreated cells (Fig. 1a, b). In response to HG, LPS and Hcy, neutrophils released significant (*p* < 0.0001) amounts of elastase levels which were similar in quantities (Fig. 1c). Immunoblotting assays of neutrophil lysates revealed that all inducers activated citrullination of histones (H3), although the strength of citrullination varied between inducers (Figs. 1d, e) where we observed prominent effects when treated with homocysteine as compared to high glucose and LPS. Interestingly, we also observed intra-individual variations in citrullinated histone levels. Probing for pan-tyrosine phosphorylation antibodies in neutrophils treated with HG, LPS and Hcy indicated no changes in overall tyrosine phosphorylation levels (Fig. S1).

### High glucose, homocysteine, and LPS significantly alter neutrophil phosphoproteome

**Fig. 2:**
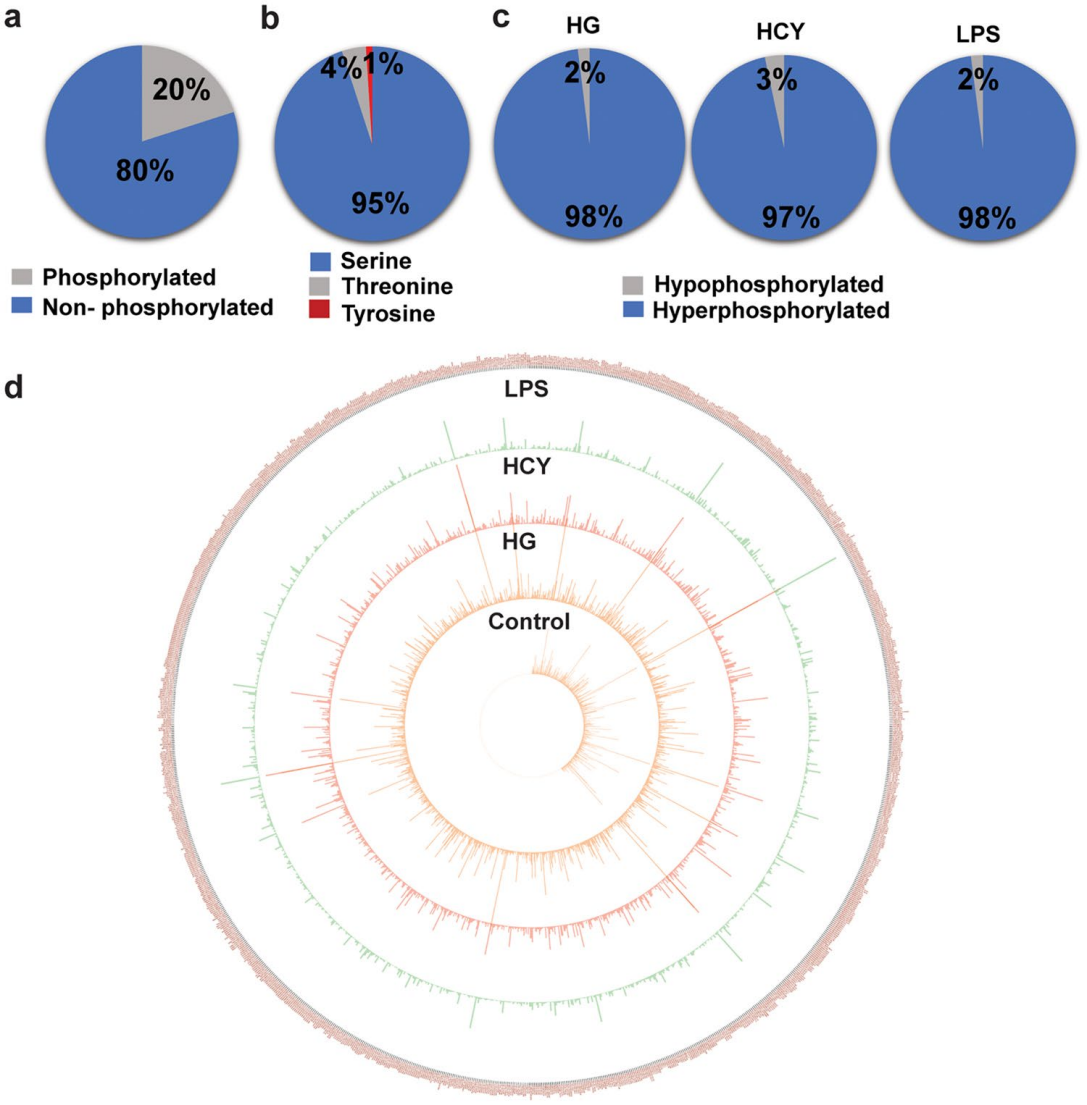
Neutrophil phosphoproteome in response to diverse inducers. (**a**) Pie graph showing total number of phosphorylated and non-phosphorylated peptides in the overall dataset. (**b**) Pie chart depicting the distribution of phosphorylation sites across differentially phosphorylated proteins. (**c**) Pie chart illustrating the distribution of differentially phosphorylated proteins of neutrophils treated with indicated inducers compared with control. (**d**) Circos diagram plotted using abundance ratios of phosphoprotein signatures obtained from neutrophils in response to HG, LPS, and Hcy along with control

We observed HG, LPS, and Hcy associated with different pathological conditions induced significant NET formation. Hence, this instigated us to examine whether these inducers stimulate distinct signaling pathways to activate neutrophils. To identify early and unique phosphorylation events, we carried out a phosphoproteomic analysis of human neutrophils treated with HG, LPS, and Hcy for 30 min. In overall phosphoproteomics dataset, we identified peptides corresponding to 6771 proteins, out of which 1361 (20%) were phosphorylated peptides (Fig. 2a). Out of 1361 phosphorylated peptides, majority represented serine phosphorylation (95%) followed by threonine phosphorylation (4%) and tyrosine phosphorylation contributed to least proportion (1%) (Fig. 2b). Further analysis revealed upon stimulation, proportions of hyperphosphorylated peptides were higher than hypophosphorylated peptides (Fig. 2c). HG treatment led to phosphorylation of 845 peptides (≥ 1.5-fold change Vs control), and 15 peptides were hypophosphorylated (≤ 0.6-fold change Vs control). Accordingly, LPS stimulation showed 902 peptides were hyperphosphorylated (≥ 1.5-fold change Vs control), and 19 peptides were hypophosphorylated (≤ 0.6 fold change Vs control). Neutrophils upon stimulation with Hcy displayed hyperphosphorylation of 844 peptides (≥ 1.5-fold change Vs control), and 29 peptides were hypophosphorylated (≤ 0.6 fold change Vs control). Subsequent analysis of 1361 phosphorylated peptides revealed significant alterations in neutrophil phosphoproteome upon activation with HG, LPS, and Hcy (Fig. 2d). Circos representation indicated that more than 60.4% of peptides were either minimally or not phosphorylated in constitutive levels and activation of neutrophils led to phosphorylation of peptides. We also observed HG, LPS, and Hcy further modulated the phosphorylation levels in stimulus-specific manner suggesting activation of inducer-dependent activation of distinct signaling pathways (Fig. 2d).

### High glucose, homocysteine, and LPS induce unique phosphoproteome signatures in neutrophils

Next, we set out to identify stimulus-specific phosphorylation of peptides and associated changes. Comparing levels of 200 most abundant hyper and hypo phosphopeptides present or absent in at least one condition led to the identification of significantly upregulated or downregulated phosphopeptides in a given condition (Fig. S2a, b). Comparing phosphopeptides obtained from HG-treated cells to that of Hcy in a color gradient heatmap revealed 76 peptides were significantly hyperphosphorylated and 112 peptides were hypophosphorylated. Further, 22 peptides were hyperphosphorylated, and 47 peptides were hypophosphorylated in HG-activated cells when compared with LPS treated. Homocysteine-induced cells showed 56 hyperphosphorylated peptides and 64 hypophosphorylated peptides in comparison to LPS. Hcy- and LPS-activated neutrophils revealed 108 and 45 hyperphosphorylated peptides, and on the other hand, 78 and 22 peptides were hypophosphorylated, respectively, when compared to HG. LPS activation led to an accumulation of 71 hyperphosphorylated and 62 hypophosphorylated peptides compared to Hcy. This indicated, neutrophils activated by different inducers displayed distinct phosphoproteome signatures.

Evaluation of localization of phosphoproteins in various cellular components upon induction of neutrophils revealed similar distribution and, however, we observed quantitative differences among phosphopeptides when treated with a different stimulus (Table 1). Majority of the phosphopeptides upon activation with HG, LPS, and Hcy harbored in intracellular membrane-bound organelle (approximately 25%) followed by the nucleus (23%), ficolin-rich granules (3.5%), actin cytoskeleton (3.5%), and focal adhesion (3%). Further, we observed many phosphopeptides activated in neutrophils were localized to azurophilic granules, specific granules, tertiary granules, and secretory granules and proportions of peptides varied among stimulation. Subcellular localization of the top ten cellular components of phosphopeptides in response to a stimulus is shown in Table 1. We further performed enrichment analysis of phosphorylated peptides by using the STRING database to identify activation of distinct signaling pathways and associated biological processes in neutrophils treated with different inducers. Global changes in phosphoproteome revealed phosphopeptides pertaining to several biological processes such as regulation of mRNA metabolic processes, granulocyte activation, neutrophil degranulation, leucocyte activation, and immune response (Fig. S3a). Interestingly, we observed glucose-, LPS-, and Hcy-induced phosphorylated peptides aligned to unique biological processes. HG-mediated phosphorylation of proteins were involved in positive regulation of intrinsic apoptotic pathway, organelle transport along microtubule, Interleukin-12 signaling pathway, toll-like receptor pathway, and leucocyte chemotaxis (Fig. S3b). Hcy-activated biological processes were different from other inducers and involved respiratory burst, negative regulation of B- cell proliferation, histone phosphorylation, and negative regulation of glucose import (Fig. S3c). Further, LPS-enriched phosphopeptides were involved in the positive regulation of mRNA splicing via spliceosomes, leucocyte degranulation and chemotaxis, phagocytosis, and Fc gamma receptor-mediated signaling pathway (Fig. S3d).

**Table 1:** (a-c) Top 10 cellular components of phosphoproteins which are enriched in HG-, Hcy-, and LPS-treated neutrophils

**a)** **High glucose**	Genes	*P* value
Intracellular membrane-bounded organelle	230/5192	6.49E-27
Nucleus	207/4484	1.02032E-25
Actin cytoskeleton	33/316	9.07171E-13
Cytoskeleton	46/600	2.20377E-12
Ficolin-1-rich granule	23/184	7.54686E-11
Focal adhesion	31/387	3.49437E-09
Ficolin-1-rich granule lumen	17/123	4.90334E-09
Cell-substrate junction	31/394	5.34892E-09
Specific granule	19/160	8.08228E-09
Intracellular non-membrane-bounded organelle	60/1158	8.21826E-09

**b)** **Homocysteine**	Gene	*P* value
Intracellular membrane-bounded organelle	218/5192	5.65883E-21
Nucleus	197/4484	1.10413E-20
Ficolin-1-rich granule	27/184	4.34328E-14
Actin cytoskeleton	32/316	6.48959E-12
Cytoskeleton	45/600	1.25764E-11
Focal adhesion	34/387	7.15329E-11
Ficolin-1-rich granule lumen	19/123	1.06259E-10
Cell-substrate junction	34/394	1.15897E-10
Secretory granule membrane	25/274	1.16063E-08
Intracellular non-membrane-bounded organelle	60/1158	1.31722E-08

**c)** **LPS**	Overlap	*P* value
Intracellular membrane-bounded organelle	243/5192	6.32741E-25
Nucleus	219/4484	3.47493E-24
Ficolin-1-rich granule	27/184	3.39293E-13
Secretory granule membrane	32/274	1.35215E-12
Cell-substrate junction	38/394	4.13161E-12
Focal adhesion	37/387	1.03528E-11
Actin cytoskeleton	33/316	1.32314E-11
Cytoskeleton	46/600	6.56184E-11
Specific granule membrane	16/91	1.53947E-09
Tertiary granule	20/164	1.08686E-08

**Fig. 3:**
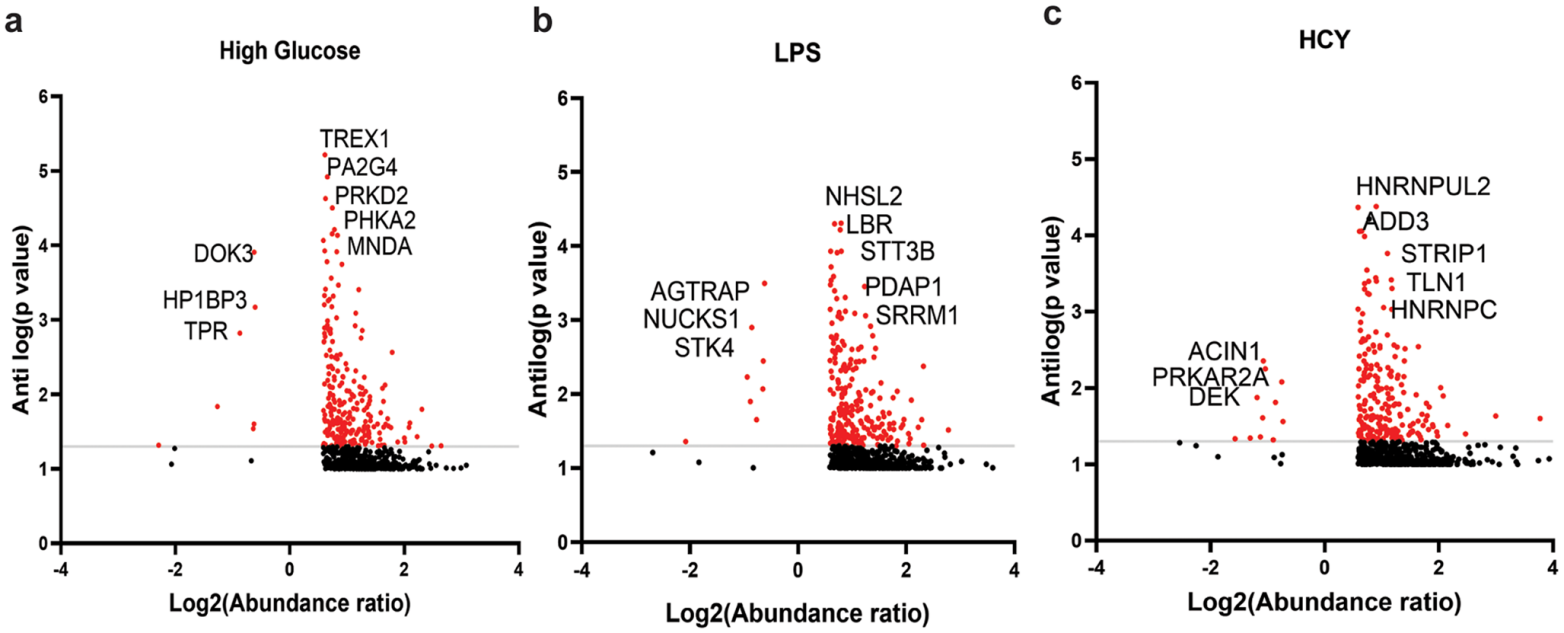
Volcano plots showing both hyper and hypophosphorylated proteins based on Log_2_ (abundance ratio) and antilog *P* value. Highly abundant proteins expressed in different conditions are indicated

Subsequently, we plotted volcano graphs to identify differentially phosphorylated peptides in neutrophils treated with different inducers (Fig. 3). In response to HG out of 644 peptides, we found 237 significantly hyperphosphorylated and 7 hypophosphorylated peptides. LPS treatment resulted in the identification of 670 peptides of which 222 phosphopeptides were significantly upregulated and 8 peptides were hypophosphorylated. Accordingly, Hcy induction led to the identification of 649 peptides where 218 peptides were significantly hyperphosphorylated and 11 peptides were hypophosphorylated.

Statistically significant and most abundant hyperphosphorylated peptides in HG-treated neutrophils were identified as TREX1, PA2G4, PHKA2, MNDA, and PRKD2 involved in biological processes such as cytosolic sensors of pathogen-associated DNA, neutrophil degranulation, and metabolism of carbohydrates and lipids (Table 2). Similarly, we found HP1BP3, DOCK3, and TPR peptides related to hypoxia and cytokine signaling pathways were hypophosphorylated in response to HG. On the other hand, LPS stimulated significant upregulation in phosphorylation of STT3B, PDAP1, SRRM1, LBR, and NHSL2 which are involved in pathways concerning epigenetic regulation, degranulation, cholesterol metabolism, and cell differentiation. We also observed LPS induced significant hypophosphorylation of AGTRAP, NUCKS1, and STK4 proteins related to pathways associated with angiotensin II receptor, DNA repair and signal transduction (Table 2). Hcy-induced hyperphosphorylation of HNRNPUL2, ADD3, TLN1, HNRNPC, and STRIP1 associated with actin binding, RNA binding, and cytoskeletal organization, and hypophosphorylated peptides included AC1N1, DEK, and PRKAR2A related to apoptosis and signal transduction (Table 2).

**Table 2:** Representative phosphopeptides uniquely found in different conditions and associated functions

Genes	Peptides of corresponding proteins	P-site	Functions
**High glucose**—Hyper-Phosphorylated peptides			
TREX1	Three-prime repair exonuclease 1 isoform b	S167(98.4)	Cytosolic sensors of pathogen-associated DNA
PA2G4	Proliferation-associated protein 2G4	S2(100)	ERBB3-regulated signal transduction pathway
PRKD2	Serine/threonine-protein kinase D2 isoform A	S197(100)	Metabolism of lipids
PHKA2	Phosphorylase b kinase regulatory subunit alpha, liver isoform X1	S7(100)	Metabolism of carbohydrates
MNDA	Myeloid cell nuclear differentiation antigen	S227(100	Neutrophil degranulation
Hypo-phosphorylated peptides			
HP1BP3	Heterochromatin protein 1-binding protein 3 isoform 1	S441(100), S442(97.9)	DNA binding, Cellular response to hypoxia
TPR	Nucleoprotein TPR	S2155(100)	Cytokine Signaling in immune system, Cellular responses to external stimuli
DOK3	Docking protein 3 isoform 4	S274(98)	Neutrophil degranulation
**LPS**—Hyper-phosphorylated peptides			
STT3B	Dolichyl-diphosphooligosaccharide–protein glycosyltransferase subunit STT3B	S498(100), S499(100)	Post-translational protein modification
PDAP1	Heat- and acid-stable phosphoprotein	S60(100)	Neutrophil degranulation, cell signaling
SRRM1	Serine/arginine repetitive matrix protein 1 isoform 24	S697(100), S699(100)	DNA and RNA binding
LBR	Delta (14)-sterol reductase LBR isoform X1	[S99(100)	Metabolic pathway leading to cholesterol biosynthesis
NHSL2	NHS-like protein 2	[S847(100)	Cell differentiation
Hypo-phosphorylated peptides			
AGTRAP	Type-1 angiotensin II receptor-associated protein isoform X3	S143(98.7)	Negative regulator of type-1 angiotensin II receptor-mediated signaling by regulating receptor internalization
NUCKS1	Nuclear ubiquitous casein and cyclin-dependent kinase substrate 1	S73(100); S79(100)	DNA repair
STK4	Serine/threonine-protein kinase 4 isoform X1	S336(100	Signal transduction
**Homocysteine**—Hyper-Phosphorylated peptides			
HNRNPUL2	Heterogeneous nuclear ribonucleoprotein U-like protein 2	S193(100)	RNA binding
ADD3	Gamma-adducin isoform X1	T12(98.1)	Signal transduction, Transportation of small molecules
STRIP1	Striatin-interacting protein 1 isoform 1	S335(100)	Regulation of cell morphology and cytoskeletal organization
TLN1	Talin-1	S1225(100)	Actin cytoskeleton organization
HNRNPC	Heterogeneous nuclear ribonucleoprotein K	S247(100)	Actin binding
Hypo-phosphorylated peptides			
AC1N1	Apoptotic chromatin condensation inducer in the nucleus isoform 1	S216(100)	Apoptosis
PRKAR2A	cAMP-dependent protein kinase type II-alpha regulatory subunit isoform X1	S78(100); S80(100	Signal transduction, Megakaryocyte development and platelet production
DEK	Protein DEK isoform X1	S301(98.2)	DNA and RNA binding, Signal transduction

### Diverse inducers activate distinct set of kinases in neutrophils

**Fig. 4:**
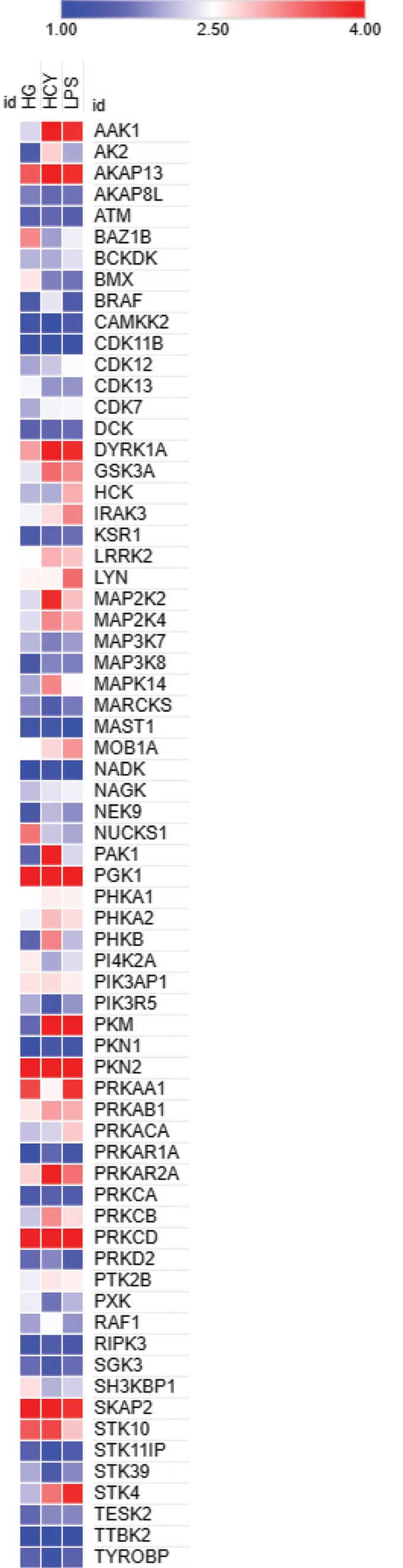
Heat map displaying differentially phosphorylated kinases in neutrophils treated with HG, LPS and Hcy comparing with control. Scale bar indicates magnitude of the phosphorylation

We identified phosphorylated states of kinases in our dataset and heatmaps plotted revealed differential phosphorylation of kinases among HG-, LPS-, and Hcy-treated cells. HG-induced hypophosphorylation of AAK1, GSK3A, PAK1, and PKM, whereas both HG and LPS hyperphosphorylated these kinases. In contrast, HG increased phosphorylation of SKAP2 as compared to other inducers. Homocysteine distinctly phosphorylated PAK1 but not in cells treated with HG and LPS. Further, LYN and STK4 kinases were phosphorylated in cells treated with LPS (Fig. 4).

**Fig. 5:**
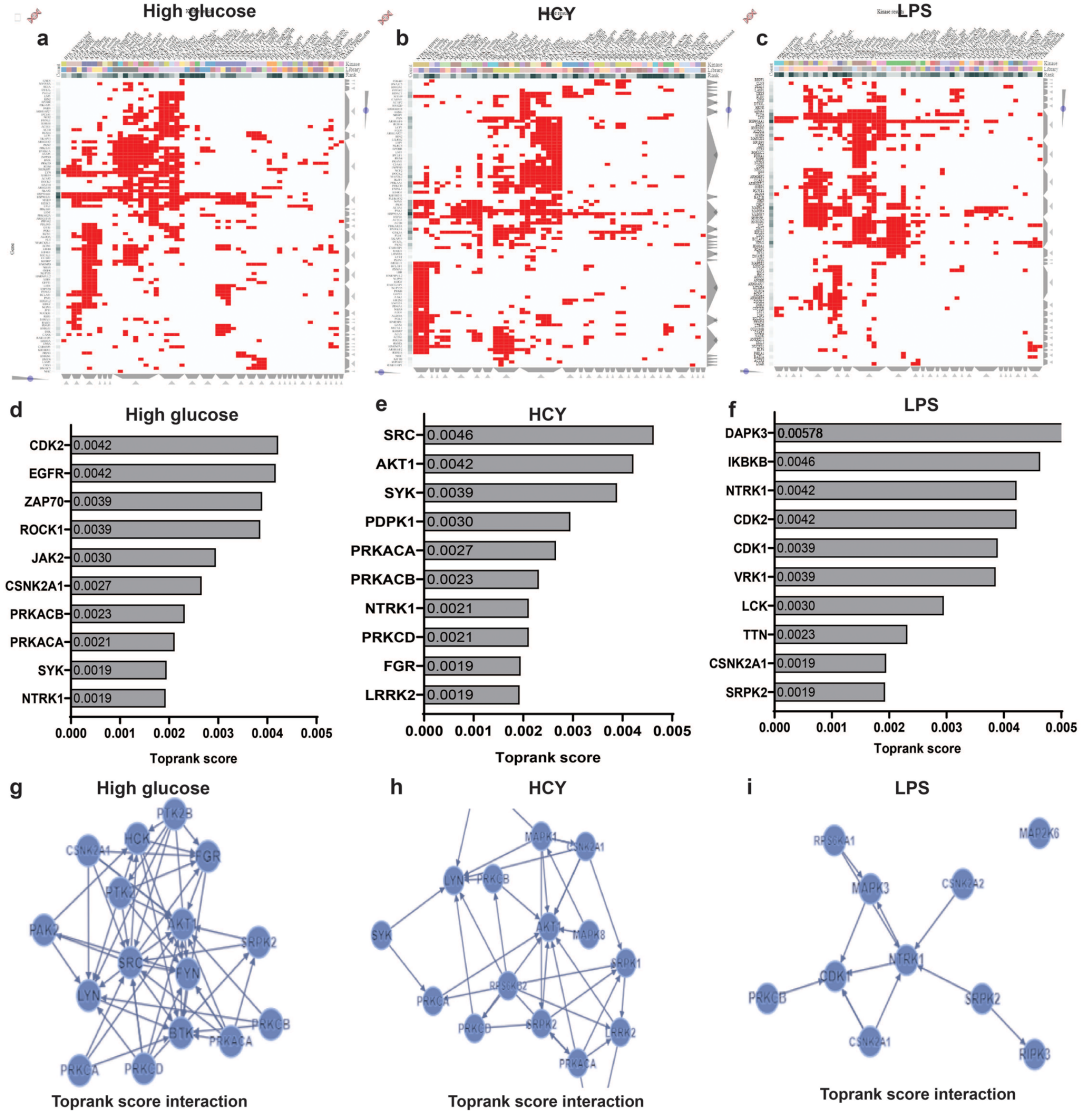
Identification of upstream kinases of phosphorylated proteins and their interactions. (**a**–**c**) Interactive heat map interpreting kinase and substrate interaction, each column represents kinases and rows represents substrate. (**d**–**f**) Bar graphs representing top rank score of top 10 kinases with integrated scaled rank along with p values in X axis. (**g**–**i**) Kinase enrichment analysis showing kinase -substrate interaction

To obtain further insights into distinct signaling pathways activated by different inducers, we looked into possible upstream kinases responsible for differential phosphorylation of peptides. Interactive heat map revealed varied patterns of clusters of substrate-kinase interaction across high glucose, homocysteine, and LPS-treated neutrophils (Fig. 5a–c). Kinome maps constructed with hyperphosphorylated peptides showed the involvement of stimulus-specific distinct sets of kinases responsible for neutrophil activation (Fig. 5d, e). Subsequent analysis revealed HG-treated neutrophils led to activation of NTRK1, SYK, and PRKACA kinases, and some of their target peptides of these kinases were OGFR, NBAS, PRKAA1, HSP90AB1, RGS14, NCF2, and ACTB. Reactome analysis revealed that these peptides were involved in Rho GTPase signaling and phagocytosis. Similarly, LPS induction resulted in activation of kinases such as SRPK2, CSNK2A1 and TTN phosphorylating peptides CTR9, HP1BP3, HNRNPU, BAZ2A, MLASP1, MAP3K7, and MAP2K4 involved in cytokine signaling and inflammatory response. Accordingly, Hcy-activated LRRK2, FGR, and PRKCD kinases responsible for phosphorylating PRAA1, NCF2, C5AR1, RIPOR2, GSK3A, and HSP90AA1 associated with neutrophil degranulation, VEGF-VEGFR2 signaling, and cytoskeletal remodeling. Taken together, our phosphoproteome data showed the activation of a distinct set of upstream kinases in response to diverse stimuli.

Subsequently, we looked into the top 20 networks of kinases based on known kinase-kinase interactions observed in neutrophils treated with high glucose, homocysteine, and LPS. High glucose treated neutrophils showed a strong interaction between kinases such as AKT, BTK, FGR, SRC and FYN, whereas in homocysteine treated cells displayed strong interaction between AKT, MAPK1, LYN, PRKCB, LLRK2, SRPK1, and RPSGKP2. Similarly, we observed strong interaction between MAPK3, NTRK1, and CDK1 kinases in treated LPS neutrophils (Fig. 5g–i).

**Fig. 6:**
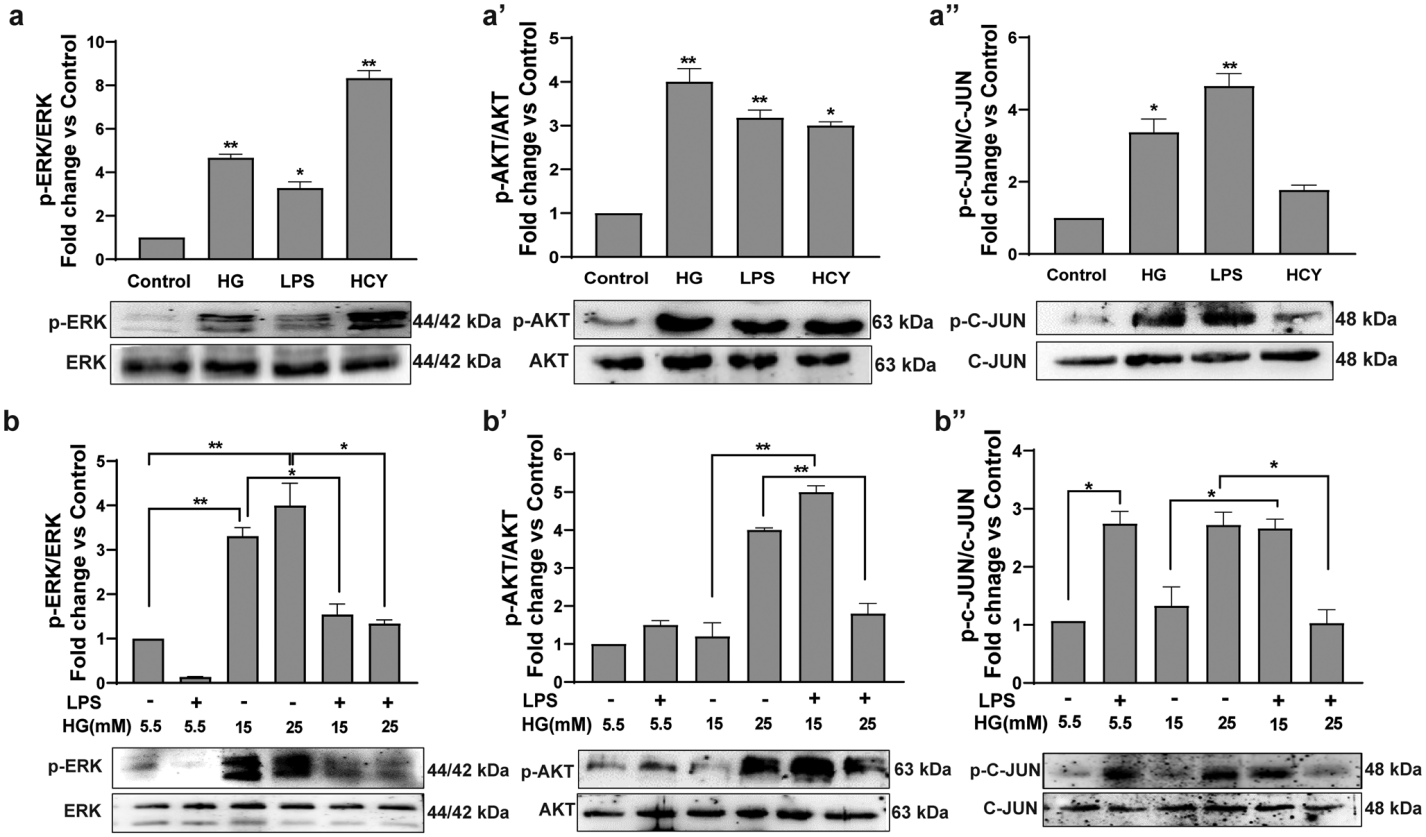
Neutrophils respond differently to diverse inducers and LPS effects are blunted in high glucose pre-treated cells (**a**, **a’**, **a’’**). Neutrophils isolated from peripheral venous blood were stimulated with high glucose (25 mM), LPS (2 µg/mL), and Hcy (250 µM) for 30 min. The neutrophil lysates were subjected to immunoblotting. Representative blots stained for phospho- ERK1/2^Thr202/Tyr204^ and total- ERK; phospho AKT^Ser473^ and total AKT (**a’**); phospho- C-JUN^Ser63^ and total C-JUN (**a’’**) are shown. (**b**, **b’**, **b’’**) Neutrophils were cultured under different glucose concentrations in the presence or absence of LPS with indicated concentrations for 3 h and further stimulated with LPS for 30 min. Cell lysates were subjected to immunoblotting. Representative blots stained for phospho- ERK and total- ERK; phospho AKT and total AKT (**b’**) and phospho- C-JUN and total C-JUN (**b’’**) are provided. Data are represented as fold change Vs control and statistically significant values are indicated by asterisk *pvalue ≤ 0.05, **p value ≤ 0.001, ***p value ≤ 0.0001

Based on phospho-proteome changes in response to various inducers and upstream kinase analysis, we further validated phosphorylation levels of ERK1/2^Thr202/Tyr204^, AKT^Ser473^, and C-JUN N-terminal Kinase^Ser63^ in neutrophils treated with HG, LPS, and Hcy for 30 min. We found all three inducers showed varied effects on the phosphorylation status of these kinases. Homocysteine induced a nearly eightfold increase in ERK1/2^Thr202/Tyr204^ phosphorylation, whereas high glucose and LPS led to approximately fourfold and threefold phosphorylation, respectively. All inducers showed similar effects on Akt^Ser473^ phosphorylation. Interestingly, both high glucose and LPS but not homocysteine induced phosphorylation of C-Jun- N-Terminal kinase^Ser63^ (Fig. 6a).

To examine the influence of LPS in T2D-conditioned neutrophils, we pre-treated neutrophils in different concentrations of glucose (5.5 mM, 15 mM, and 25 mM) for 3 h and subsequently stimulated with LPS for 30 min. We observed HG significantly upregulated phosphorylation of ERK1/2^Thr202/Tyr204^ after 3 h, and inclusion of LPS reduced the effects of HG. However, LPS alone did not induce ERK1/2 phosphorylation. Further, 25-mM glucose induced threefold increase in Akt^Ser473^ phosphorylation, and these effects were abrogated upon LPS stimulation. LPS and HG concentrations increased phosphorylation of C-Jun-N-Terminal Kinase^Ser63^, and LPS effects were blunted in HG-pretreated neutrophils. Taken together, we observed T2D-conditioned neutrophils showed impeded response to LPS (Fig. 6b).

### Neutrophils display differential phosphorylation of granular proteins associated with a various cellular process in response to diverse inducers

**Fig. 7:**
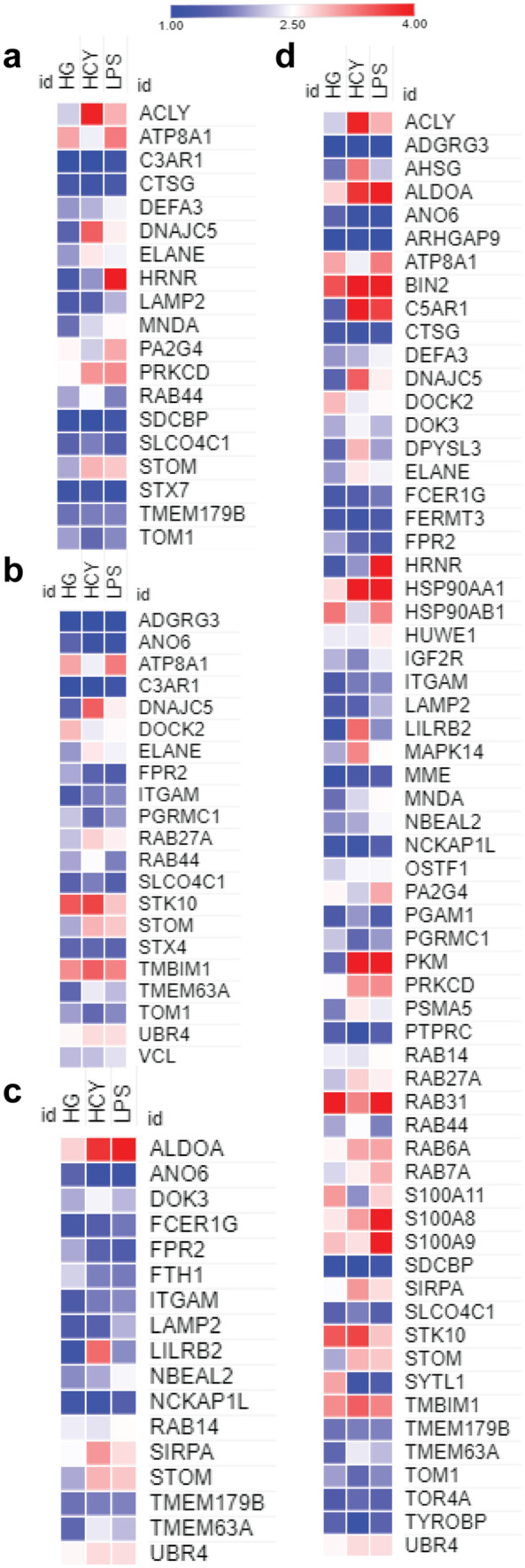
Heat map visualization of the phosphorylated proteins localized in different granules in response to high glucose, LPS, and homocysteine. Differentially modulated phosphoproteins in (**a**) azurophilic granules, (**b**) secondary granules, (**c**) tertiary granules, and (**d**) secretory granules are provided. Scale bar shows magnitude of the phosphorylation

Neutrophils, belonging to granulocyte lineage contain four different types of coexisting granules in the cytoplasm: (a) azurophilic, (b) specific, (c) tertiary, and (d) secretory ( Borregaard et al. 2007). These granules harbor microbicidal proteins and facilitate in eliminating infections. Based on earlier literature, we cataloged neutrophil-specific proteins and granular proteins (Rørvig et al. 2013; Adrover et al. 2020) and examined whether these proteins were differentially phosphorylated in response to HG, Hcy, and LPS (Fig. 7). Color gradient heat map construction indicated azurophilic granular proteins such as ACLY, DNAJC5, and STOM were abundantly hyperphosphorylated in Hcy-treated neutrophils, whereas HRNR and PA2G4 phosphorylation were enriched in LPS stimulated cells (Fig. 7a). Analysis of specific/secondary granular proteins showed DOCK2 was highly phosphorylated in HG-treated cells, whereas DNAJC5, RAB27A, and STK10 were profusely phosphorylated in Hcy-treated neutrophils (Fig. 7b). Tertiary granular proteins such as LILRB2, SIRPA, and STOM were significantly phosphorylated in Hcy-treated cells compared to other conditions (Fig. 7c). Further HG-induced neutrophils showed a highly phosphorylated state of secretory granular proteins such as DOCK2, SYTL1, and S100A11, whereas ACLY, AHSG, DNAJC5, LILRB2, and SIRPA were highly expressed in Hcy-induced cells. Similarly, LPS-treated cells contained highly phosphorylated secretory granular proteins such as S100A8, S100A9, PA2G4, and HRNR proteins (Fig. 7d). Taken together, this suggested neutrophils treated with HG, Hcy, and LPS led to phosphorylation of a unique set of granular proteins involved in various functions.

**Fig. 8:**
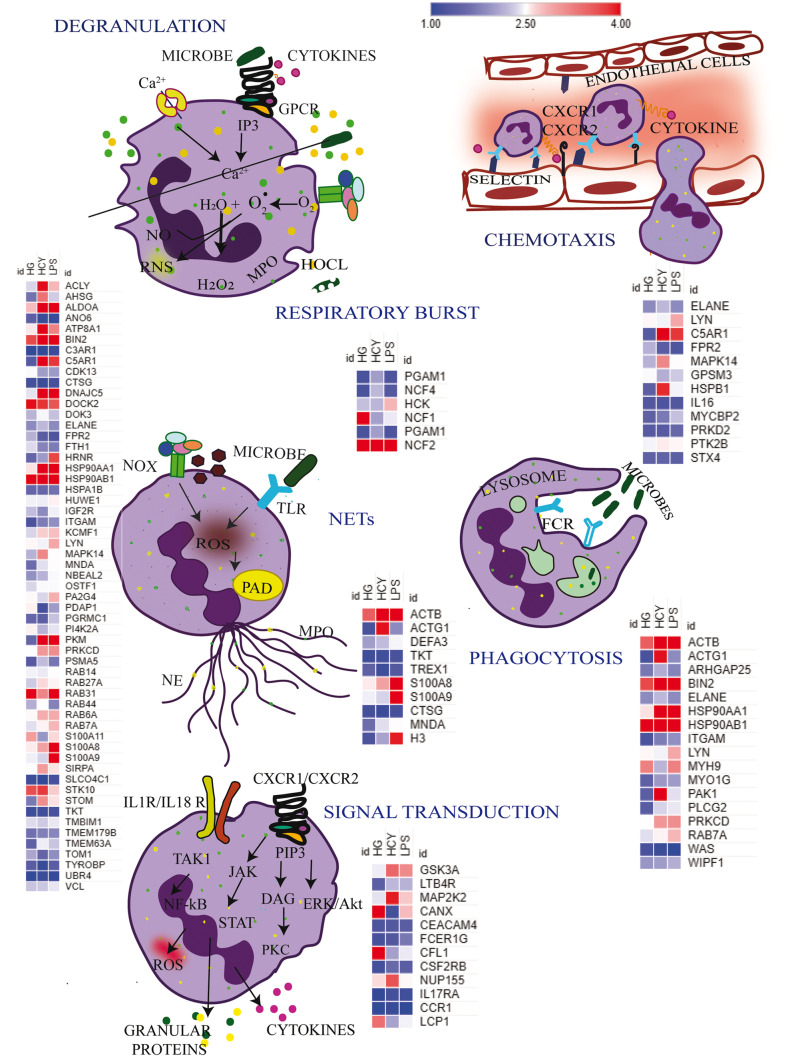
Phosphoproteome reprogramming in neutrophils in response to different inducers and associated functions. Heat map representing phosphorylation pattern of proteins involved in diverse neutrophil functions upon treatment with high glucose, LPS, and Hcy. Scale bar shows magnitude of the phosphorylation

Next, we stratified proteins involved in different functions of neutrophils such as chemotaxis, degranulation, respiratory burst, phagocytosis, and NETosis and examined differential phosphorylation of these proteins in response to HG, Hcy, and LPS (Fig. 8). Proteins associated with diverse signaling pathways associated with neutrophil functions displayed differential phosphorylation in presence of inducers. GSK3A, MAP2K2, and NUP155 were significantly phosphorylated in response to Hcy and CANX; CFL1 and LCP1 were hyperphosphorylated in cells treated with HG. We observed proteins involved in chemotaxis such as LYN phosphorylation was higher in response to LPS, whereas HSPB1 and MAPK14 were hyperphosphorylated when cells were treated with Hcy. C5AR1 was phosphorylated in cells treated with Hcy and LPS but not when treated with HG. Proteins associated with neutrophil degranulation showed significant differences among inducers. LPS stimulation led to hyperphosphorylation of S100A9, S100A8, HRNR, and PA2G4 but not by HG and Hcy. ACLY, MAPK14, and STK10 were hyperphosphorylated by Hcy. Phosphorylation of HSP90AA1, ALDOA, C5AR1, DNAJC5, and PKM was increased in Hcy- and LPS-treated cells but not in cells activated by HG. RAB31 phosphorylation was increased by HG and LPS. Phosphorylation of HSP90AB1, BIN2, and DOCK2 was observed in all conditions. Respiratory burst-related proteins such as HCK were significantly phosphorylated by LPS and Hcy treatment led to phosphorylation of NCF1. ACTG1 and PAK1 associated with phagocytosis were significantly phosphorylated by Hcy alone. HSP90AA1 and PRKCD phosphorylation was observed upon stimulation with Hcy and LPS but not by HG, while ACTB, BIN2, and HSP90AB1 phosphorylation was observed in all conditions. NET-associated proteins such as S100A8, S100A9, and H3 showed differential phosphorylation among inducers where LPS-mediated effects were more pronounced.

## Discussion

The purpose of our study was to understand upstream signaling events during neutrophil activation in response to clinically relevant stimulants in T2D and associated complications that were not explored earlier. Hence, we examined influence of HG, Hcy, and LPS on neutrophil phosphoproteome changes. We identified a variety of distinct phosphoprotein signatures corresponding to upstream kinases and early signaling intermediates along with granular proteins in response to HG, Hcy, and LPS. HG-induced phosphorylation of proteins is involved in Rho GTPase signaling and phagocytosis, whereas Hcy treatment led to the activation of pathways associated with neutrophil degranulation and cytoskeletal remodeling. Further, LPS-induced pathways are involved in cytokine signaling and inflammatory response. These differentially phosphorylated proteins also aligned to neutrophil functions including degranulation, oxidative burst, phagocytosis, and NETosis. Validation of phosphoproteomics data obtained in our analysis on selected kinases revealed neutrophils pre-cultured under high glucose showed impeded response to LPS to phosphorylate p-ERK1/2^Thr202/Tyr204^, p-AKT^Ser473^, and C-Jun-N-Terminal Kinase^Ser63^ kinases.

In recent years, several studies have explored proteomics-based approaches to decipher underlying cellular mechanisms regulating neutrophil functions. Comparative proteomics of neutrophil supernatants containing NETs in response to various inducers showed diverge proteome composition in LPS compared to that of PMA- and A23187-induced NETs (Petretto et al. 2019). Using enrichment analysis, authors showed proteins identified in NETs induced by A23187 aligned to calcium signaling and LPS and PMA induced signaling pathways related to JAK/STAT signaling and interleukins (Petretto et al. 2019). Shotgun proteomics analysis of neutrophils from T2D subjects of good and poor glycemic controls revealed 35 differentially expressed protein components of NETs (Soongsathitanon et al. 2019). Authors observed neutrophils from T2D subjects with poor glycemic controls displayed downregulation of myeloperoxidase, S100A9, and azurocidin and upregulation of glycolytic enzymes such as alpha-enolase and transketolase (Soongsathitanon et al. 2019). Bruschi et al. (2020) identified distinct protein fingerprints and post-translational modifications on proteins in NETs formed in subjects with serum lupus erythematosus (SLE) and lupus nephritis (Bruschi et al. 2020). Quantitative proteomics analysis of NETs in response to PMA and A23187 in neutrophils from rheumatoid arthritis and SLE indicated PMA-induced NETs contained proteins of annexin family, azurocidin, and histone H3, whereas A23187 enriched CAMP/LL37, CRISP3, lipocalin, IL-8, and PADI4 (Chapman et al. 2019). Comparative phosphoproteomics analysis on neutrophils treated with PMA, ionomycin, and monosodium urate showed differential phosphorylation of proteins involved in pathways associated with nuclear function, chromatin binding, and RNA binding activities (Zhu and Chen 2019). Proteomics analysis in rodent neutrophils revealed an intrinsic program to degranulate in steady state, thereby reducing NETs forming ability and subsequently decreasing magnitude of inflammation under physiological conditions (Adrover et al. 2020). Authors, further demonstrated progressive degranulation at night upon enrichment of granular proteins such as S100A9, S100A8, CTSG, ACTN1, and MPO and this process was temporally regulated by CXCR2, protecting from excessive inflammation and vascular damage (Adrover et al. 2020). Our data revealed differential phosphorylation of proteins such as HSP90AA1, ACLY, DNAJC5, HRNR, RAB31, S100A8, and S100A9 associated with degranulation process in response to Hcy and HG which may lead to altered and excessive neutrophil degranulation, subsequently triggering vascular damage in diseases such as T2D and atherosclerosis.

Hyper activation of neutrophils and constitutive NETosis is one of the major pathological consequences in T2D and is associated with recurrent infections and thrombosis. Neutrophils upon activation induce stimulus-specific kinases and associated signaling intermediates to form extracellular traps (Kenny et al. 2017). In response to various microbes and their products, signaling pathway activators/inhibitors and immune complexes neutrophils produce extracellular traps upon activating upstream kinases such as MAPK/ERK (MEK), IL-1 receptor-associated kinase (IRAK), protein kinase C (PKC), phosphoinositide 3 kinase (PI3K), and AKT/PKB (Gabriel et al. 2010; Saitoh et al. 2012; Douda et al. 2014; Behnen et al. 2014; Papayannopoulos 2018). LPS induces NETs through TLR-4 dependent and JNK-mediated phosphorylation of NOX2 (Khan et al. 2017). PMA, a pharmacological activator of protein kinase C induces NET formation via Raf-MEK-ERK signaling axis and in NOX2-dependent manner ( Hakkim et al. 2011). Leishmania induced ROS-dependent and independent NETosis via PI3Kγ/ERK and calcium/PI3Kδ, respectively (DeSouza‐Vieira et al. 2016). Monosodium urate crystals promoted ROS-independent NETs formation via receptor-interacting serine/threonine-protein kinase 1 (RIPK1) and RIPK3 (Desai et al. 2016). In the context of T2D pathology, high glucose induced ERK1/2 mediated translocation of NADPH oxidase subunit and constitutive generation of superoxides in diabetic neutrophils (Omori et al. 2008). Taken together, along with kinases identified in earlier studies, we also found novel kinases and substrates involved in several physiological and pathological processes mediated by neutrophils.

Mounting pieces of evidence indicate that neutrophils display significant heterogeneity in both phenotype and functions and respond diversely to physiological and pathological stimuli (Kolaczkowska and Kubes 2013; Yvan-Charvet and Ng 2019). Neutrophils perform diverse functional responses including phagocytosis, oxidative burst, and degranulation; produce extracellular traps; and regulate adaptive immune responses (Nauseef and Borregaard 2014). Single-cell RNA sequencing analysis in mouse neutrophils have also recently shown complex and heterogeneous subpopulations of neutrophils under steady-state and emergency granulopoiesis (Xie et al. 2020). Taken together, this indicates neutrophils upon responding to diverse physiological and pathological stimuli induce distinct signaling pathways resulting in specific functions. Interestingly, our phospho-proteomics analysis of neutrophils in response to various pathological stimuli in the context of T2D revealed differential phosphorylation of peptides/proteins corresponding to diverse functions of neutrophils. As described earlier, previous proteomics analyses have identified a variety of neutrophil proteins including granular proteins, histones, and S100 family proteins in both healthy and inflammatory states and their functional relevance. However, earlier studies have performed either unlabeled descriptive or semi-quantitative proteomics and identified proteins at endpoint of NET formation. Hence, our study might serve as a resource for early neutrophil phosphoproteome signatures and facilitate in understanding mechanisms regulating paradoxical effects of neutrophils in diseases such as T2D. However, validating key differentially phosphorylated proteins corresponding to important biological processes in in vivo and clinical models of T2D with or without (a) infections and (b) thrombosis is warranted. Subsequent studies may help in designing therapeutic strategies upon inhibiting glucose-induced effects and simultaneously restore NETs in the presence of infections.

## Supplementary information

Below is the link to the electronic supplementary material.

Supplementary file1 (DOCX 2961 KB)

## Data Availability

The mass spectrometry proteomics data have been deposited to the ProteomeXchange Consortium via the PRIDE partner repository with the dataset identifier PXD029046.

## References

[CR1] Adrover JM, Aroca-Crevillén A, Crainiciuc G, Ostos F, Vega YR, Ponce AR, Cilloniz C, Bonzón-Kulichenko E, Calvo E, Rico D, Moro MA, Weber C, Lizasoaín I, Torres A, Ruiz-Cabello J, Vázquez J, Hidalgo A (2020). Programmed ‘disarming’ of the neutrophil proteome reduces the magnitude of inflammation. Nat Immunol.

[CR2] Behnen M, Leschczyk C, Möller S, Batel T, Klinger M, Solbach W, Laskay T (2014). Immobilized immune complexes induce neutrophil extracellular trap release by human neutrophil granulocytes via FcγRIIIB and Mac-1. J Immunol.

[CR3] Borregaard N, Sørensen OE, Theilgaard-Mönch K (2007). Neutrophil granules: a library of innate immunity proteins. Trends Immunol.

[CR4] Brinkmann V, Reichard U, Goosmann C, Fauler B, Uhlemann Y, Weiss DS, Weinrauch Y (2004). Neutrophil Extracellular Traps Kill Bacteria. Science.

[CR5] Bruschi M, Bonanni A, Petretto A, Vaglio A, Pratesi F, Santucci L, Migliorini P, Bertelli R, Galetti M, Belletti S, Cavagna L, Moroni G, Franceschini F, Fredi M, Pazzola G, Allegri L, Sinico RA, Pesce G, Bagnasco M, Manfredi A, Ramirez GA, Ramoino P, Bianchini P, Puppo F, Pupo F, Negrini S, Mattana F, Emmi G, Garibotto G, Santoro D, Scolari F, Ravelli A, Tincani A, Cravedi P, Volpi S, Candiano G, Ghiggeriet GM (2020). Neutrophil extracellular traps profiles in patients with incident systemic lupus erythematosus and lupus nephritis. J Rheumatol.

[CR6] Chapman EA, Lyon M, Simpson D, Mason D, Beynon RJ, Moots RJ, Wright HL (2019). Caught in a trap? Proteomic analysis of neutrophil extracellular traps in rheumatoid arthritis and systemic lupus erythematosus. Front Immunol.

[CR7] De Meyer SF, Suidan GL, Fuchs TA, Monestier M, Wagner DD (2012). Extracellular chromatin is an important mediator of ischemic stroke in mice. Arterioscler Thromb Vasc Biol.

[CR8] Desai J, Kumar SV, Mulay SR, Konrad L, Romoli S, Schauer C, Herrmann M, Bilyy R, Müller S, Popper B, Nakazawa D, Weidenbusch M, Thomasova D, Krautwald S, Linkermann A, Anders HJ (2016). PMA and crystal-induced neutrophil extracellular trap formation involves RIPK1-RIPK3-MLKL signaling. Eur J Immunol.

[CR9] DeSouza-Vieira T, Guimarães-Costa A, Rochael NC, Lira MN, Nascimento MT, Lima-Gomez PDS, Mariante RM, Persechini PM, Saraiva EM (2016). Neutrophil extracellular traps release induced by Leishmania: role of PI3Kγ, ERK, PI3Kσ, PKC, and Ca2+. J Leukoc Biol.

[CR10] Díaz-Godínez C, Carrero JC (2019) The state of art of neutrophil extracellular traps in protozoan and helminthic infections. Biosci Rep 39(1):BSR20180916 10.1042/BSR20180916 PMC6328873 30498092

[CR11] Douda DN, Yip L, Khan MA, Grasemann H, Palaniyar N (2014). Akt is essential to induce NADPH-dependent NETosis and to switch the neutrophil death to apoptosis. Blood.

[CR12] Gabriel C, McMaster WR, Girard DCG, Descoteaux A (2010). Leishmania donovani promastigotes evade the antimicrobial activity of neutrophil extracellular traps. J Immunol.

[CR13] Gan YH (2013). Host Susceptibility Factors to Bacterial Infections in Type 2 Diabetes. PLoS Pathog.

[CR14] Hair PS, Echague CG, Rohn RD, Krishna NK, Nyalwidhe JO, Cunnion KM (2012). Hyperglycemic conditions inhibit C3-mediated immunologic control of Staphylococcus aureus. J Transl Med.

[CR15] Hakkim A, Fuchs TA, Martinez NE, Hess S, Prinz H, Zychlinsky A, Waldmann H (2011). Activation of the Raf-MEK-ERK pathway is required for neutrophil extracellular trap formation. Nat Chem Biol.

[CR16] Joshi MB, Ahamed R, Hegde M, Nair AS, Ramachandra L, Satyamoorthy K (2020). Glucose induces metabolic reprogramming in neutrophils during type 2 diabetes to form constitutive extracellular traps and decreased responsiveness to lipopolysaccharides. Biochim Biophys Acta Mol Basis Dis.

[CR17] Joshi MB, Baipadithaya G, Balakrishnan A, Hegde M, Vohra M, Ahamed A, Nagri SK, Ramachandra L, Kapaettu Satyamoorthy K (2016). Elevated homocysteine levels in type 2 diabetes induce constitutive neutrophil extracellular traps. Sci Rep.

[CR18] Joshi MB, Lad A, Bharath Prasad AS, Balakrishnan A, Ramachandra L, Satyamoorthy S (2013). High glucose modulates IL-6 mediated immune homeostasis through impeding neutrophil extracellular trap formation. FEBS Lett.

[CR19] Kaplan MJ, Radic M (2012). Neutrophil Extracellular Traps: Double-Edged Swords of Innate Immunity. J Immunol.

[CR20] Kenny EF, Herzig A, Krüger R, Muth A, Mondal S, Thompson PR, Brinkmann V, Bernuth HV, Zychlinsky A (2017). Diverse stimuli engage different neutrophil extracellular trap pathways. Elife.

[CR21] Khan MA, Farahvash A, Douda DN, Licht JC, Grasemann H, Sweezey N, Palaniyar N (2017). JNK Activation Turns on LPS- and Gram-Negative Bacteria-Induced NADPH Oxidase-Dependent Suicidal NETosis. Sci Reports.

[CR22] Kolaczkowska E, Kubes P (2013). Neutrophil recruitment and function in health and inflammation. Nat Rev Immunol.

[CR23] Kuwabara WMT, Panveloski-Costa AC, Yokota CNF, Pereira JNB, Filho JM, Torres RP, Hirabara SM, Curi R, Loureiroet TCA (2017). Comparison of Goto-Kakizaki rats and high fat diet-induced obese rats: Are they reliable models to study Type 2 Diabetes mellitus?. PLoS ONE.

[CR24] Menegazzo L, Ciciliot S, Poncina N, Mazzucato M, Persano M, Bonora B, Albiero M, Kreutzenberg SVD, Avogaro A, Fadini GP (2015). NETosis is induced by high glucose and associated with type 2 diabetes. Acta Diabetol.

[CR25] Najar MA, Aravind A, Dagamajalu S, Sidransky D, Ashktorab H, Smoot DT, Gowda H, Prasad TK, Modi PK, Chatterjee A (2021a) Hyperactivation of MEK/ERK pathway by Ca 2+ /calmodulin-dependent protein kinase kinase 2 promotes cellular proliferation by activating cyclin-dependent kinases and minichromosome maintenance protein in gastric cancer cells. Mol Carcinog 60(11):769–783 10.1002/mc.23343 34437731

[CR26] Najar MA, Modi PK, Ramesh P, Sidransky D, Gowda H, Prasad TS, Chatterjee H (2021). Molecular Profiling Associated with Calcium/Calmodulin-Dependent Protein Kinase Kinase 2 (CAMKK2)-Mediated Carcinogenesis in Gastric Cancer. J Proteome Res.

[CR27] Nauseef WM, Borregaard N (2014). Neutrophils at work. Nat Immunol.

[CR28] Omori K, Ohira T, Uchida Y, Ayilavarapu S, Batista BL, Yagi M, Iwata T, Liu H, Hasturk H, Kantarci A, Dyke TEV (2008). Priming of neutrophil oxidative burst in diabetes requires preassembly of the NADPH oxidase. J Leukoc Biol.

[CR29] Papayannopoulos V (2018). Neutrophil extracellular traps in immunity and disease. Nat Rev Immunol.

[CR30] Petretto A, Bruschi M, Pratesi F, Croia C, Candiano G, Ghiggeri G, Migliorini P (2019). Neutrophil extracellular traps (NET) induced by different stimuli: A comparative proteomic analysis. PLoS ONE.

[CR31] Rørvig S, Østergaard O, Heegaard NH, Borregaard N (2013). Proteome profiling of human neutrophil granule subsets, secretory vesicles, and cell membrane: correlation with transcriptome profiling of neutrophil precursors. J Leukoc Biol.

[CR32] Rosales C (2018). Neutrophil: A cell with many roles in inflammation or several cell types?. Front Physiol.

[CR33] Saitoh T, Komano J, Saitoh Y, Misawa T, Takahama M, Kozaki T, Uehata T, Iwasaki H, Omori H, Yamaoka S, Yamamoto N, Akira S (2012). Neutrophil extracellular traps mediate a host defense response to human immunodeficiency virus-1. Cell Host Microbe.

[CR34] Schuetz P, Castro P, Shapiro NI (2011). Diabetes and sepsis: preclinical findings and clinical relevance. Diabetes Care.

[CR35] Soongsathitanon J, Umsa-Ard W, Thongboonkerd V (2019). Proteomic analysis of peripheral blood polymorphonuclear cells (PBMCs) reveals alteration of neutrophil extracellular trap (NET) components in uncontrolled diabetes. Mol Cell Biochem.

[CR36] Stegenga ME, van der Crabben SN, Blümer RM, Levi M, Meijers JCM, Serlie MJ, Tanck HMWT, Sauerwein HP, Poll TVD (2008). Hyperglycemia enhances coagulation and reduces neutrophil degranulation, whereas hyperinsulinemia inhibits fibrinolysis during human endotoxemia. Blood.

[CR37] Verma R, Pinto SM, Patil AH, Advani J, Subba P, Kumar M, Sharma J, Dey G, Ravikumar R, Buggi S, Satishchandra P (2017) Quantitative proteomic and phosphoproteomic analysis of H37Ra and H37Rv strains of mycobacterium tuberculosis. J Proteome Res 16:1632–1645 10.1021/acs.jproteome.6b00983 28241730

[CR38] Wong SL, Demers M, Martinod K, Gallant M, Wang Y, Goldfine AB, Kahn CR, Wagner DD (2015). Diabetes primes neutrophils to undergo NETosis which severely impairs wound healing. Nat Med.

[CR39] Xie X, Shi Q, Wu P, Zhang X, Kambara H, Su J, Yu H, Park SY, Guo R, Ren Q, Zhang S, Xu Y, Silberstein LE, Cheng T, Ma F, Li C, Luo HR (2020). Single-cell transcriptome profiling reveals neutrophil heterogeneity in homeostasis and infection. Nat Immunol.

[CR40] Yang S, Gu Z, Lu C, Zhang T, Guo X, Xue G, Zhang L (2020). Neutrophil extracellular traps are markers of wound healing impairment in patients with diabetic foot ulcers treated in a multidisciplinary setting. Adv Wound Care.

[CR41] Yvan-Charvet L, Ng LG (2019). Granulopoiesis and neutrophil homeostasis: a metabolic, daily balancing act. Trends Immunol.

[CR42] Zhu X, Chen J (2019). Phosphoproteomic analyses provide insight into molecular mechanisms underlying NETosis. Proteomics.

