# Supplementary material for: Quantitative phosphoproteomics reveals diverse stimuli activate distinct signaling pathways during neutrophil activation
Source: Cell Tissue Res. 2022 May 27;389(2):241–57. doi: 10.1007/s00441-022-03636-7 (PMC9287233; doi:10.1007/s00441-022-03636-7)
Supplement: Supplementary file 1 — Supplementary file1 (DOCX 2961 KB) [file 441_2022_3636_MOESM1_ESM.docx]

**Quantitative Phosphoproteomics Reveals Diverse Stimuli Activate Distinct Signaling Pathways During Neutrophil Activation**

**Journal of Cell Communication and Signaling**

Pooja Yedehalli Thimmappa^1^, Aswathy S Nair^1^, Mohd. Altaf Najar ^2^, Varshasnata Mohanty ^2^, Shamee Shastry ^3^, Thottethodi Subrahmanya Keshava Prasad ^2^, Manjunath B Joshi*^1^

^1^ Department of Ageing Research, Manipal School of Life Sciences, Manipal Academy of Higher Education, Manipal, 576104, India

^2^ Center for Systems Biology and Molecular Medicine, Yenepoya Research Centre, Yenepoya (Deemed to be University), Mangalore 575020, India

^3^Department of Immunohematology and Blood Transfusion, Kasturba Medical College, Manipal Academy of Higher Education, Manipal, 576104, India

***Corresponding Author**

Dr. Manjunath B Joshi

Associate Professor

Manipal School of Life Sciences

Planetarium Complex

Manipal Academy of Higher Education

Manipal-576104

India

Tel-+91 820 2922058

Fax-+91 820 2571919

Electronic address: manjunath.joshi@manipal.edu


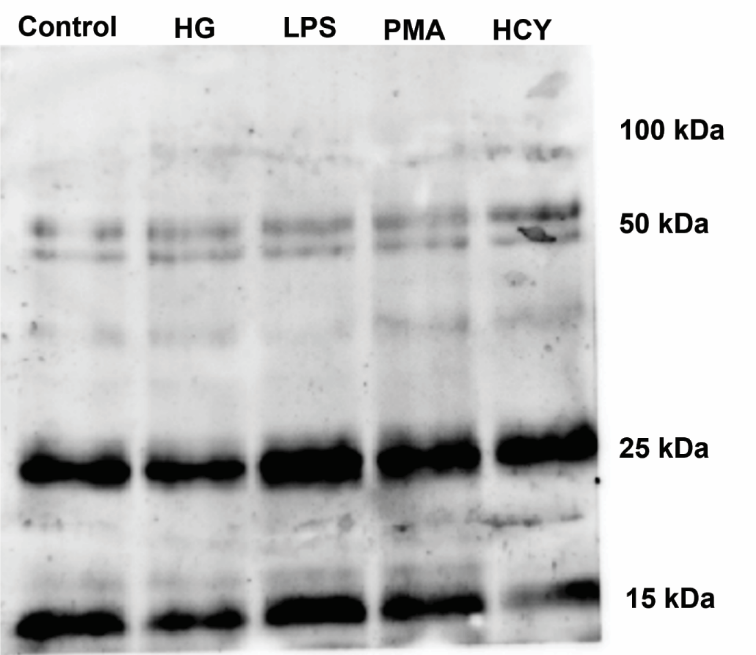


**Fig S1** Neutrophils were treated with high glucose, LPS, PMA and homocysteine for 30 minutes and lysed. Cell lysates were processed for immunoblotting and probed for phospho-tyrosine antibody.


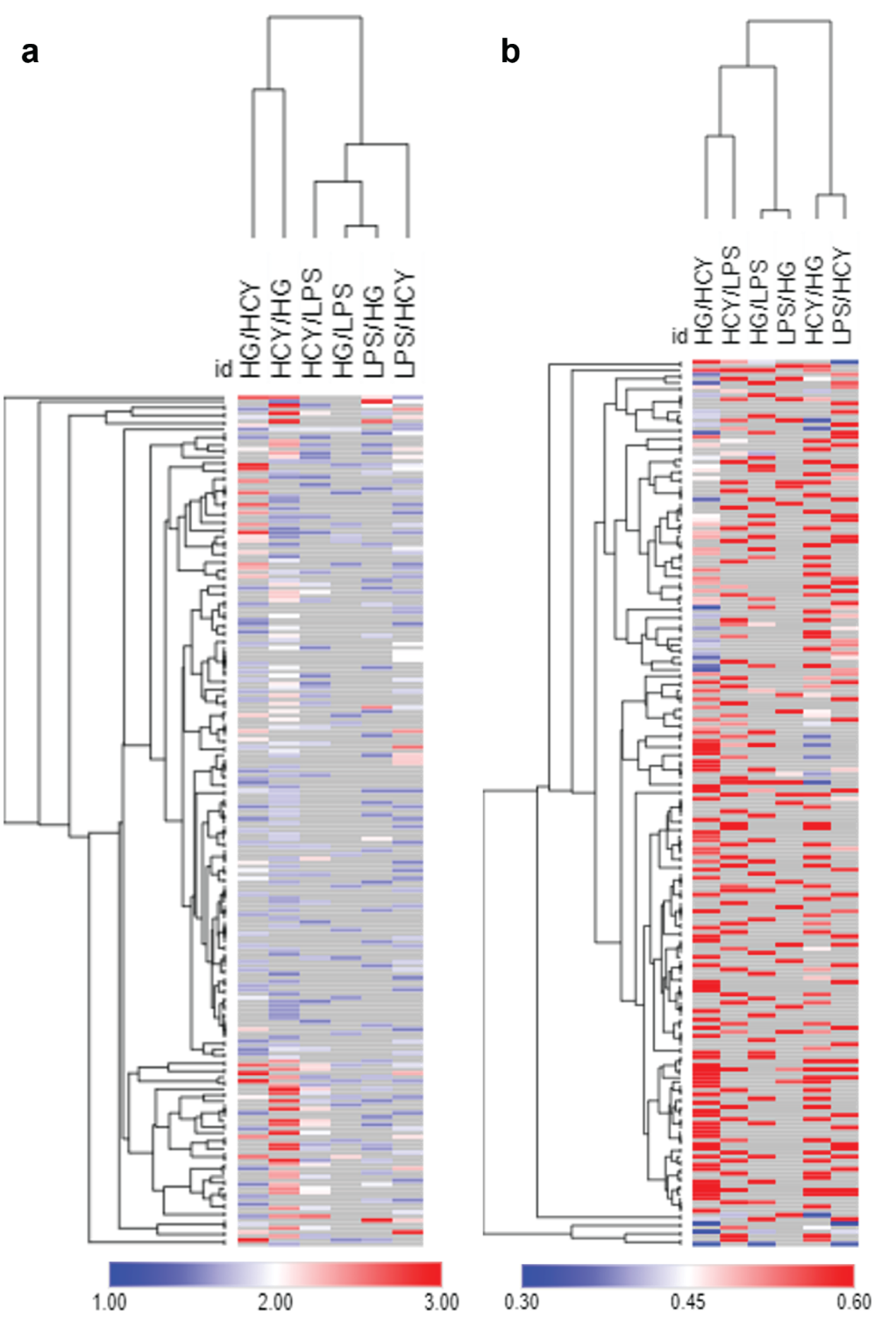


**Fig S2** Hierarchical clustering of differentially expressed phosphorylated peptides in any one of the conditions in neutrophils treated with High glucose (HG), LPS, homocysteine (HCY). a) Differentially expressed hyperphosphorylated peptides, b) hypophosphorylated peptides. Scale bar showing the magnitude of the phosphorylation.


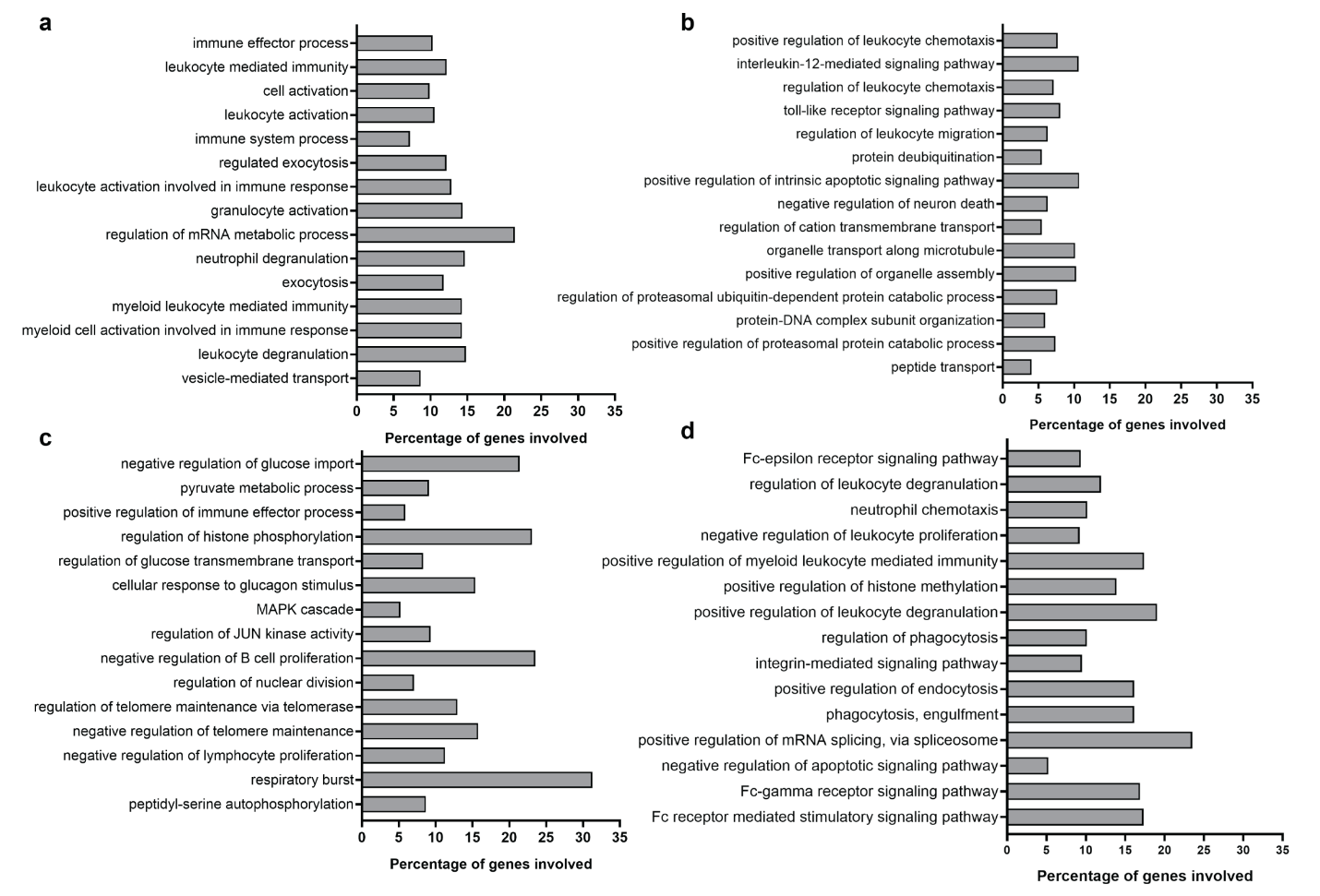


**Fig S3** Gene Ontology indicating biological processes enriched in (a) overall proteins obtained in the phosphoproteomic analysis of Neutrophils, and treated with (b) High glucose, (c) homocysteine and (d) LPS.


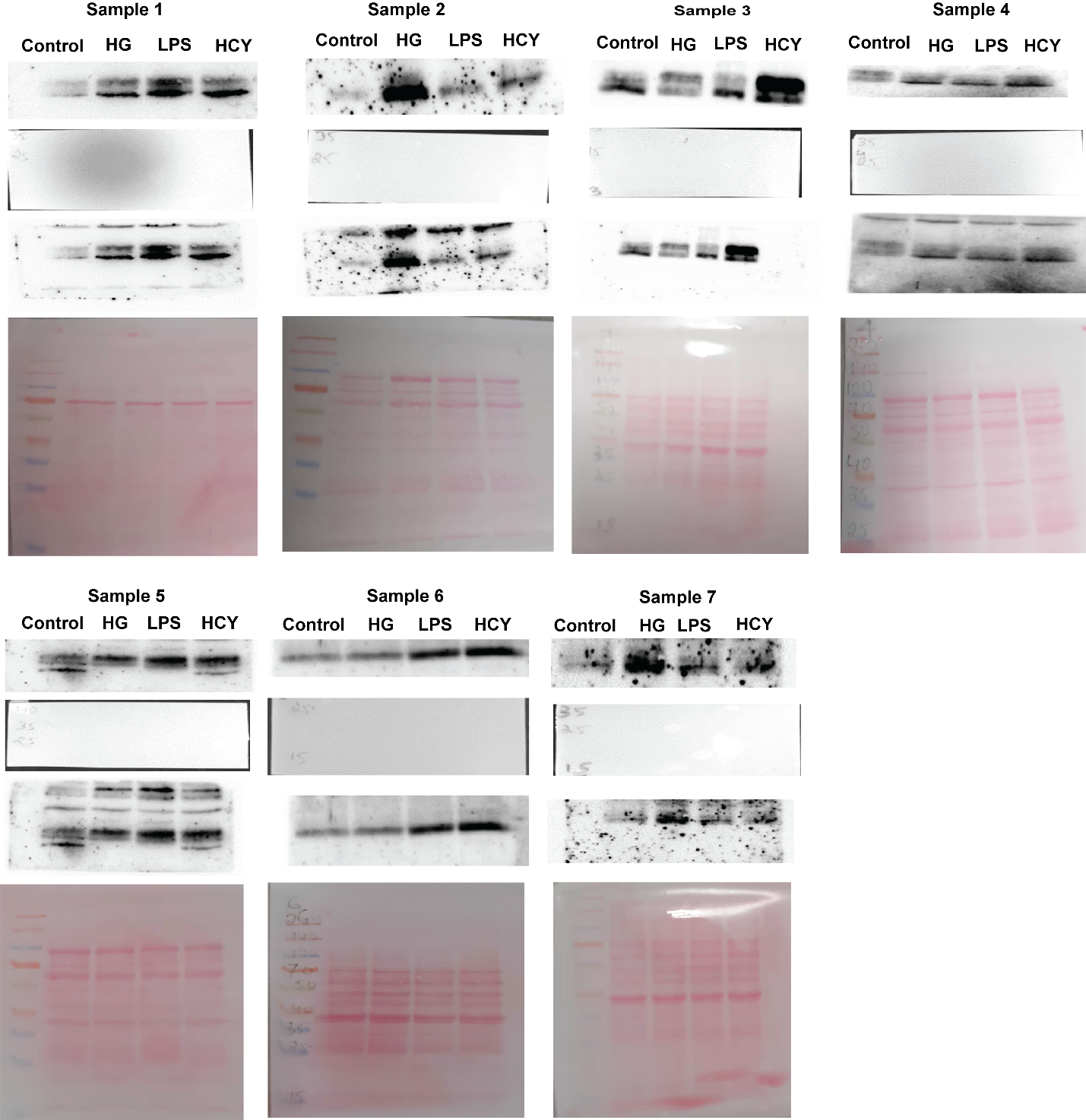


**Fig S4** Raw data for immunoblots for citrullinated histone levels in different individuals.
